# Resolution of Uncontrolled Type 2 Diabetes after Laparoscopic Truncal Vagotomy, Subtotal Gastrectomy, and Roux-en-Y Gastrojejunostomy for a Patient with Intractable Gastric Ulcers

**DOI:** 10.1155/2012/102752

**Published:** 2012-11-03

**Authors:** Laura F. Tait, Gezzer Ortega, Daniel D. Tran, Terrence M. Fullum

**Affiliations:** ^1^Howard University College of Medicine, Washington, DC 20059, USA; ^2^Division of Bariatric and Minimally Invasive Surgery, Howard University Hospital, Washington, DC 20060, USA

## Abstract

*Background*. Laparoscopic Roux-en-Y gastric bypass (LRYGB) has been shown to be an effective treatment for type 2 diabetes mellitus (T2DM) in patients with morbid obesity. However, it is unclear just how effective the LRYGB procedure is on T2DM for patients with BMI less than 35 kg/m^2^. We report one obese patient with T2DM who did not meet the current NIH criteria for morbid obesity surgery. This patient underwent a laparoscopic truncal vagotomy, subtotal gastrectomy, and Roux-en-Y gastrojejunostomy for intractable gastric ulcers and subsequently had full resolution of her T2DM. *Methods*. A 48-year-old patient with a BMI of 34.6 kg/m^2^ underwent a laparoscopic truncal vagotomy, subtotal gastrectomy, and Roux-en-Y gastrojejunostomy for intractable gastric ulcers. The patient was seen 3 months preoperatively, followed for 24 months postoperatively, and evaluated for postoperative complications, weight loss, and improvement in comorbidities. *Results*. The patient had no postoperative surgical complications. Her BMI decreased from 34.6 kg/m^2^ to 22.3 kg/m^2^ by 24 months postoperatively. Significant improvements in her fasting blood glucose levels were seen 10 days postoperatively from a preoperative level of 147 mg/dl to 97 mg/dl. *Conclusion*. Patients with a BMI less than 35 kg/m^2^ and uncontrolled T2DM may benefit from a laparoscopic Roux-en-Y gastric bypass.

## 1. Introduction 

Obesity is a growing epidemic and is strongly associated with an increase in the prevalence of comorbid conditions, including type 2 diabetes mellitus (T2DM), cardiovascular disease, and cancer. In the United States, the development of T2DM has been strongly linked to obesity, with 50% of T2DM patients having a BMI >30 kg/m^2^ [1]. T2DM is now an epidemic, accounting for 90–95% of all cases of diabetes mellitus and affecting more than 246 million people worldwide. This number is expected to increase to 380 million people by the year 2025 [2, 3]. In the United States, the most common complications to long-standing, uncontrolled T2DM include heart disease, myocardial infarction, stroke, vision loss, renal failure, and peripheral artery disease often times resulting in amputations. While current medical therapies for T2DM can reduce the incidence of complications, they have not been effective in providing a definitive cure [4]. Bariatric surgery has been shown to be the most effective therapy in the resolution of comorbid conditions including T2DM in obese patients [5]. 

The role of surgical therapy as the primary management of T2DM is promising but controversial. The mechanism of action has been studied, but there remains an unclear explanation as to what postsurgical effects are the most important in alleviating the disease [6]. Resolution of T2DM can be explained by changes in adipose tissue, leptin levels, and decreased stress on the intracellular endoplasmic reticulum by weight loss. Yet, significant improvement in glycemic control after bariatric surgery in patients with diabetes often precedes major weight loss through a weight-independent mechanism [2]. The malabsorptive surgical weight loss procedures have been proven to be the most effective in restoring euglycemia in obese patients. Roux-en-Y gastric bypass (RYGB), a common surgical weight loss procedure that is both restrictive and malabsorptive, has shown particular promise in the management of T2DM [2] by offering a potential cure. 

The role of RYGB as a long-lasting and effective procedure for controlling T2DM in the morbidly obese is well defined. Currently, under the NIH guidelines, bariatric surgery is indicated when a patient's body mass index (BMI) is greater than 40 kg/m^2^ or greater than 35 kg/m^2^ with life-threatening comorbidities such as T2DM [6]. Only a few studies have investigated RYGB as being useful in the control of T2DM in the obese patient whose BMI is less than 35. Shah et al. 2009 reported the benefit of using Roux-en-Y gastric bypass (RYGB) in a prospective study involving 15 Asian Indian patients whose BMIs were less than 35 kg/m^2^. They achieved 80% remission of T2DM 1 month postoperatively and 100% euglycemia at 3-month followup [7]. We investigated one T2DM patient with a BMI lower than 35 who underwent a procedure very similar to a laparoscopic RYGB.

## 2. Case Report

The patient is a 48-year-old Caucasian female who underwent laparoscopic truncal vagotomy, subtotal gastrectomy, and Roux-en-y gastrojejunostomy for gastric and duodenal ulcers unresponsive to medication. Past medical history was significant for intractable gastric and peptic ulcer disease for 3 years, type 2 diabetes mellitus (T2DM), hypertension, hypercholesterolemia, gastritis, esophagitis, gastroparesis, and obesity. Her BMI was 34.6 kg/m^2^. She had a Zollinger-Ellison syndrome workup, which was negative. Her T2DM medications included Lantus 30 U once a day, Januvia 100 mg once a day, and Metformin 1000 mg twice daily. Three months prior to surgery, her HbA1c was 7.9% and fasting glucose was 147 mg/dL. 

**Figure 1:**
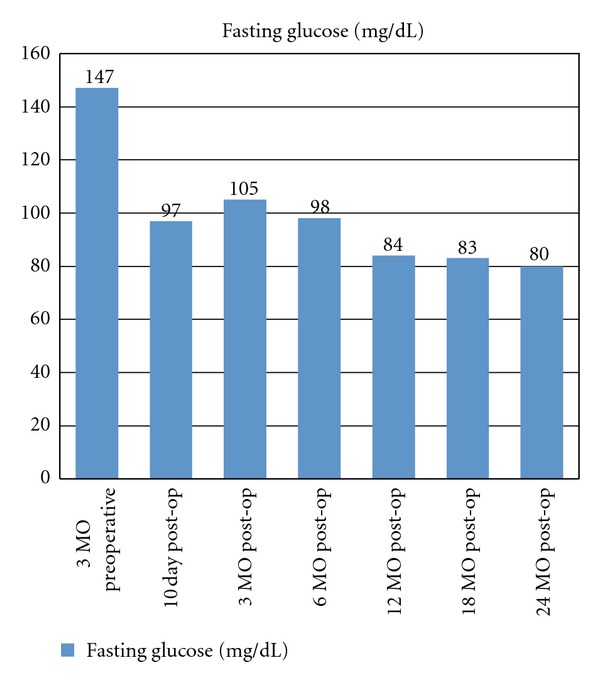
Patient's fasting glucose 3 months preoperatively through 24 months postoperatively.

**Figure 2:**
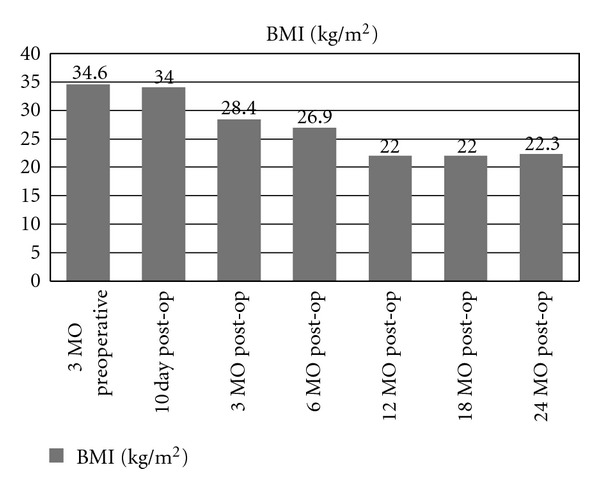
Patient's BMI 3 months preoperatively through 24 months postoperatively.

The patient underwent an uncomplicated laparoscopic truncal vagotomy, subtotal gastrectomy, and Roux-en-Y gastrojejunostomy. A subtotal gastrectomy was performed instead of an antrectomy because of her history of gastroparesis and multiple gastric ulcers. She had a small duodenal ulcer as well. Within 10 days of her bariatric procedure, rapid improvements in her fasting blood glucose levels, HbA1c, as well as her weight were noted. Her blood glucose, HbA1c, and BMI on day 10 postoperatively were 97 mg/dL, 7.2%, and 34.6 kg/m^2^, respectively. At her 2-month evaluation, her HbA1c had markedly improved to 6.5% and her BMI was 29.2 kg/m^2^. She remained off all insulin and hypoglycemic agents after her 10-day postoperative visit. Within 6 months, the patient's BMI had improved to 26.9 kg/m^2^ and she had a HbA1c of 6.1% (Figure 2). The only medication currently prescribed at that time was Metoprolol for her blood pressure. At one-year followup, the patient had blood glucose levels within the normal range (below 100 mg/dL) and was discharged from her endocrinologist due to resolution of her T2DM. The patient has had no further need of insulin or oral medications for blood sugar control since 10 days postoperatively, and her serum glucose values remained normal at her 18- and 24-month postoperative assessment (Figure 1). 

## 3. Discussion

**Figure 3:**
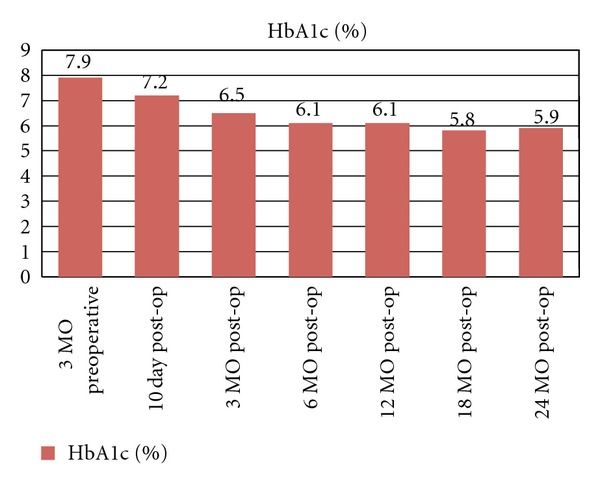
Patient's HbA1C levels 3 months preoperatively through 24 months postoperatively.

Type II diabetes mellitus, a chronic and potentially fatal illness, may be improved with strict diet adherence, weight loss, and drug regimen, but optimal control or resolution of the disease is rarely achieved using these methods. Bariatric surgery has been successful in the treatment of type II diabetes mellitus. Recent studies have shown that bariatric surgery has antidiabetic effects with the normalization of serum glucose levels following bariatric surgery being measured long before any significant weight loss is observed. There is evidence that shows that greater than 80% of patients who undergo RYGB have a complete sustained remission of their type 2 diabetes mellitus [8]. The patient in our case review had complete resolution of her T2DM within six months of her surgery and neither required oral hypoglycemic nor insulin therapy after postoperative day 10. Her HbA1c values achieved percentages of 6.1 at 6 months and 5.9 at her 2-year followup (Figure 3).

Currently, there are few reports on the laparoscopic surgical treatment of patients with T2DM and a BMI lower than 35. The first report was by DePaula et al. in 2008. In his retrospective cohort study DePaula found that “weight loss was not a reliable predictor of (T2DM) resolution or glucose control” in postgastrectomy patients. He reported 39 patients with a BMI less than 35 who underwent either a laparoscopic ileal interposition procedure associated with a sleeve gastrectomy or a laparoscopic ileal interposition procedure associated with a diverted sleeve gastrectomy. The inclusion criteria for the study specified T2DM patients whose disease had been diagnosed for at least 3 years; documentation of HbA1c exceeding 7.5% for at least 3 months; stable weight, defined as no significant change (>3%) over the 3 months before enrollment; evidence of stable treatment with oral hypoglycemic therapy or insulin for at least 12 months. All patients had a BMI less than 35 kg/m^2^. In fact, the mean BMI was 30.1 kg/m^2^. Of the 39 patients included in the study, 86.9% achieved adequate glycemic control defined as a HbA1c <7% during a mean follow-up period of 7 months [8]. One significant finding in this paper was that zero patients required insulin therapy postoperatively. DePaula also reported that the mean percentage of weight loss was 22% and the mean postoperative BMI was 24.9 kg/m^2^. The remarkable findings of adequate glucose control, independent of weight loss, led DePaula to conclude that gastrectomy seemed to be a promising procedure for the control of T2DM.

Caloric restriction, weight loss, and hormonal as well as anatomical changes are some of the major explanations offered as possible mechanisms for improvement in glucose metabolism following surgery. However, the rapid improvement of DM after RYGB counters the argument for weight loss and caloric restriction as these methods require time to achieve proper glycemic control [2]. Instead, hormonal changes driven by anatomical rearrangement appear to be a more likely theory [3]. In T2DM, the effects of the incretin hormones, gastric inhibitory peptide (GIP) and glucagon like peptide (GLP), are impaired. GIP enhances the early phase (0–20 min) insulin response to glucose. In addition, GLP enhances both the early and late phases (20–120 min) of the response of insulin to glucose. Not only is there insulin resistance in T2DM, but there is also a loss of the early phase insulin secretion leading to persistent hyperglycemia secondary to the inability to suppress both glucagon secretion and hepatic glucose output. Other documented effects of GLP include a proliferative effect on B cells as well as delayed gastric emptying. 

Following RYGB, the effects of GLP are enhanced leading to marked improvements in both glucose metabolism as well as insulin resistance [8]. This could explain why our patient had rapid glycemic improvements immediately following surgery.

## 4. Conclusions

Currently, the role of RYGB as a weight loss procedure in patients with at least one comorbidity is reserved for patients with a BMI of 35 kg/m^2^ or greater. However, patients with uncontrolled T2DB with BMIs less than 35 should be considered for RYGB as definitive treatment of their T2DM. The NIH guidelines should be reevaluated to consider RYGB in patients with BMI <35 who have uncontrolled T2DM. More prospective studies for the use of RYGB in patients with BMI less than 35 kg/m^2^ with comorbidities should be conducted using larger sample sizes with longer follow-up evaluation to include 2 years.
